# Transcriptome Analysis of Near-Isogenic Lines Provides Novel Insights into Genes Associated with Seed Low-Temperature Germination Ability in Maize (*Zea mays* L.)

**DOI:** 10.3390/plants11070887

**Published:** 2022-03-25

**Authors:** Xuhui Li, Hairui Hu, Xinmin Hu, Guihua Wang, Xuemei Du, Li Li, Feng Wang, Junjie Fu, Guoying Wang, Jianhua Wang, Riliang Gu

**Affiliations:** 1 Beijing Innovation Center for Crop Seed Technology, Ministry of Agriculture and Rural Affairs, Key Laboratory of Crop Heterosis Utilization, Ministry of Education, College of Agronomy and Biotechnology, China Agricultural University, Beijing 100193, China; ylws2201@163.com (X.L.); wzdwqr@163.com (H.H.); hxm8612@163.com (X.H.); wangguihua1991@sina.com (G.W.); duxuemei@cau.edu.cn (X.D.); lili2016@cau.edu.cn (L.L.); wangfeng911117@sina.com (F.W.); 2 Institute of Nanfan & Seed Industry, Guangdong Academy of Science, Guangzhou 510316, China; 3 Institute of Crop Sciences, Chinese Academy of Agricultural Sciences, Beijing 100081, China; fujunjie@caas.cn (J.F.); wangguoying@caas.cn (G.W.)

**Keywords:** maize, low temperature germination ability, transcriptome sequencing, near-isogenic lines

## Abstract

Maize originated from tropical regions and is extremely sensitive to low temperature during germination. Our previous work identified a major QTL, *qp1ER1-1*, for low temperature germination ability (LTGA) of maize. Here, we introgressed *qp1ER1-1* from the tolerant line L220 into the sensitive line PH4CV to generate two near isogenic lines NIL^220-3^ and NIL^220-25^. When germinated under cold temperature for 25 days (Cold-25), the NILs showed similar seedling root length and shoot length to L220, but significantly higher than PH4CV. However, when germinated under cold temperature for 15 days (Cold-15) or under normal temperature (25 °C) for 3 days (CK-3), all lines showed similar seedling performance, indicating that introgression of *qp1ER1-1* from L220 into PH4CV could improve LTGA of NIL^220-3^ and NIL^220-25^. The whole seedlings, including root and shoot, of Cold-15 and CK-3 were harvested for transcriptome analysis, when both stayed at a similar developmental stage. Dry seed embryo was sequenced as a non-germination control (CK-0). Compared with PH4CV, the tolerant line (L220, NIL^220-3^, and NIL^220-25^) specifically expressed genes (different expressed genes, DEGs) were identified for CK-0, Cold-15, and CK-3. Then, DEGs identified from Cold-15, but not from CK-0 or CK-3, were defined as tolerant line specifically expressed LTGA genes. Finally, 1786, 174, and 133 DEGs were identified as L220, NIL^220-3^, and NIL^220-25^ specifically expressed LTGA genes, respectively. Of them, 27 were common LTGA genes that could be identified from all three tolerant lines, with two (*Zm00001d031209* and *Zm00001d031292*) locating in the confidence interval of *qp1ER1-1*. In addition, GO analysis revealed that L220 specifically expressed LTGA genes were majorly enriched in the cell division process and plasma membrane related categories. Taken together, these results provided new insight into the molecular mechanism of maize seed LTGA and facilitated the cloning of the *qp1ER1-1* gene.

## 1. Introduction

Maize (*Zea mays* L.) is one of the most important grain crops with a total production of more than one billion tons, accounting for ~30% of the world’s food supply in 2020 (https://www.statista.com). As a tropical and subtropical region originated crop, maize is extremely sensitive to cold stress, particularly at germination stage [1]. Cold-sensitivity limits maize to be grown in high-latitude regions, where the growing seasons are only 4–5 months, which requires crops to be sowed at early spring. Low temperature in early spring always imposes cold stress to maize during the germination stage, which normally results in low seedling emergence and seedling uniformity [2,3]. Thus, temperature is the most limiting factor for early planting in temperate regions [4,5]. For example, in Northeast China, cold damage occurs every 3–4 years, and maize production losses can reach 20~30% in the most severe cold-weather years [6,7]. Therefore, improving low temperature germination ability (LTGA) of maize through breeding approaches could benefit to its productivity at high-latitude regions.

Plant low temperature acclimation is a complex process. In recent decades, numerous genes have been identified as participating in overcoming the adverse impact of low temperature on seed germination. These genes were majorly associated with membrane function and cell cycle regulation [8,9,10,11,12].

The plasma membrane system of dry seed embryos was often damaged by the dehydration process during later seed development stage [13,14], and it should be repaired at early germination stage (imbibition stage) to achieve a successful germination. As low temperature slows down the speed and efficiency of membrane repair, transcripts involved in this repair played a vital role for seed LTGA [1].

Meanwhile, cell cycle, the series of molecular events that allows the cell to duplicate via mitosis, is another important pathway involved in plant LTGA [15]. Seed maturation involves inhibition of cell cycle by an increase in ABA level; while a decrease in ABA level promotes seed germination by activating a G1/S kinase to accelerate the cell cycle. These cell-cycle-related genes change their expression in response to low temperature treatment [16]. In addition, genes delaying radicle expansion, weakening endosperm, and enhancing expression of transcription factors were also reported to participate in regulating seed LTGA [17].

Transcriptome analysis is a powerful tool facilitating the identification of genes with their expression levels changing among different samples, which is of growing importance in understanding how altered expression contributes to a complex trait. Many studies have been published to show the effectiveness of transcriptome analysis in identification of new genes or regulatory pathways for plant response to low temperature stress [18,19]. By comparing cold-responsive transcriptomes of wild type and mutants or cultivars of contrasting cold tolerance, a number of genes involved in multiple biology processes, such as cell cycle, zeatin biosynthesis, hormone signal transduction, membrane part, and response stimulus, have been found to be regulated by cold stress in rice [20,21,22]. When cold stress was imposed on seedlings of maize, numerous cold-stress-related genes were also identified, and these genes fall into similar functional categories as rice research indicated [1,23,24,25,26,27,28,29]. Furthermore, stress gradient treatment revealed similar GO categories involved in the response to cold stress in maize seedling [30]. Even thousands of cold responsive genes have been identified; most were detected from seedlings and little was done for germination samples [23,24,25,26,27,28,29,30].

In previous research, we developed an F_2:3_ population by crossing a cold germination tolerant line 220 (hereafter namely L220) and a sensitive line PH4CV, to identify a major QTL (*qp1ER1-1*) for seed LTGA [31]. Here, we developed two near-isogenic lines (NILs) containing the favorable allele of *qp1ER1-1* from L220 (donor parent) in PH4CV (recruitment parent) background. The two NILs and their parents were germinated at low temperature to harvest seedlings for transcriptome sequencing, which aimed to explore the germination transcriptome involved in seed LTGA and mine the promising target genes regulated by *qp1ER1-1*.

## 2. Results

### 2.1. Characteristics of qp1ER1-1 NILs

**Figure 1:**
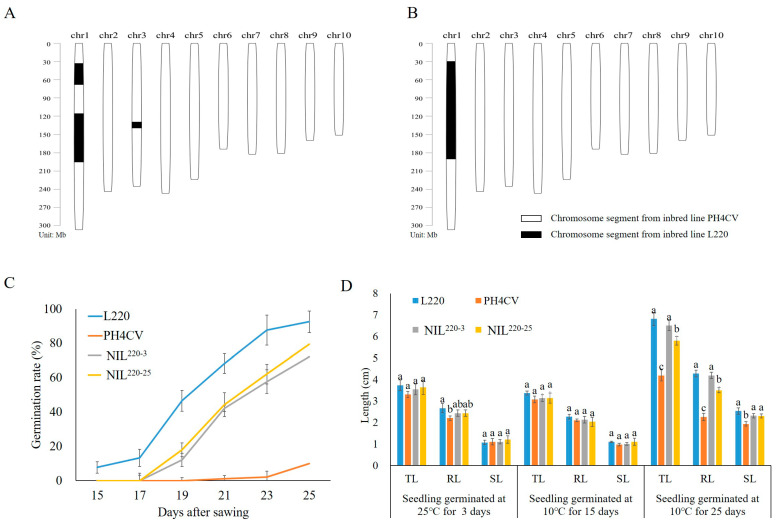
Characteristics of L220, PH4CV, and their near-isogenic lines NIL^220-3^ and NIL^220-25^. (**A**,**B**) Genomic composition of NIL^220-3^ (**A**) and NIL^220-25^ (**B**). Blank represents regions homozygous with PH4CV, and black represents homozygous regions from L220. (**C**) Emergence rate of seed germinated at 10 °C for 15–25 days. (**D**) Total length (TL), root length (RL), and shoot length (SL) of seedling germinated at 25 °C for 3 days, and at 10 °C for 15 and 25 days. Values represent mean ± SE (*n* = 3). Different letters indicate significant difference at *p* ≤ 0.05 within a trait among different genotypes that germinated in the same condition.

In our previous work, we collected two inbred lines contrasting in seed LTGA, with higher line L220 and lower line PH4CV [31]. PH4CV was a paternal parent of XY335, a widely cultivated hybrid developed by the Pioneer Technology Co., Tieling, China [32]. L220 was collected from northeast China with an untraceable pedigree [31]. In general, seed germination speed reduces when germination temperature decreases. For maize, it was reported that the minimum temperature for germination is 10 °C [33], and it was commonly set as 10 °C for the low temperature germination treatment [31,34,35]. We previously used this experimental condition to conduct QTL mapping in an F_2:3_ population crossed from L220 and PH4CV and identify a major QTL, *qp1ER1-1,* for maize LTGA [31]. Here, we backcrossed the *qp1ER1-1* allele from the cold-tolerant line L220 into the sensitive line PH4CV via marker-assisted backcrossing procedure. Two BC_4_F_4_ lines were selected for genotyping analysis, which showed that NIL^220-3^ contained three chromosome segments from the L220 genome, with one (~50 Mb length) harboring *qp1ER1-1*, and NIL^220-25^ only contained a 160 Mb segment that contained *qp1ER1-1* (Figure 1A,B).

Seed LTGA of NIL^220-3^ and NIL^220-25^ and their parent L220 and PH4CV were measured under normal (25 °C) and cold temperature (10 °C) conditions (Figure 1C,D). The two NILs had a significantly higher emergence rate (ER) than PH4CV, but slightly lower than L220 under the cold condition. Under the normal condition, all lines showed similar ER, suggesting that introgression of *qp1ER1-1* from L220 into PH4CV could increase ER of the NILs (Figure 1C).

Seedling performance traits of root length (RL), shoot length (SL), and total length (TL) in NIL^220-3^ and NIL^220-25^ were similar to those in L220, but significantly higher than those in PH4CV, when seeds germinated at cold temperature (10 °C) for 25 days (Cold-25). However, when seeds germinated at cold temperature (10 °C) for 15 days (Cold-15) and at normal temperature (25 °C) for 3 days (CK-3), no significant difference for seedling performance could be observed among these genotypes, with an exception for RL at CK-3. These results confirmed that introgression of *qp1ER1-1* in NIL^220-3^ and NIL^220-25^ could improve seed LGTA in maize, particularly at later germination stage.

### 2.2. Transcriptome Analysis for Seed LGTA

As transcript changes always occur a little earlier than the phenotypic appearances in organisms, seedlings of Cold-15 were harvested for transcriptome analysis of cold germination, where the phenotypic differences among the four genotypes did not appear, but after 10 days (at Cold-25) the differences were significant. Seedlings of CK-3 served as a normal temperature germination control, because this seedling stayed at a similar developmental stage as the Cold-15 seedling (Figure 1D). Dry embryo additionally served as a non-germination control (CK-0).

**Figure 2:**
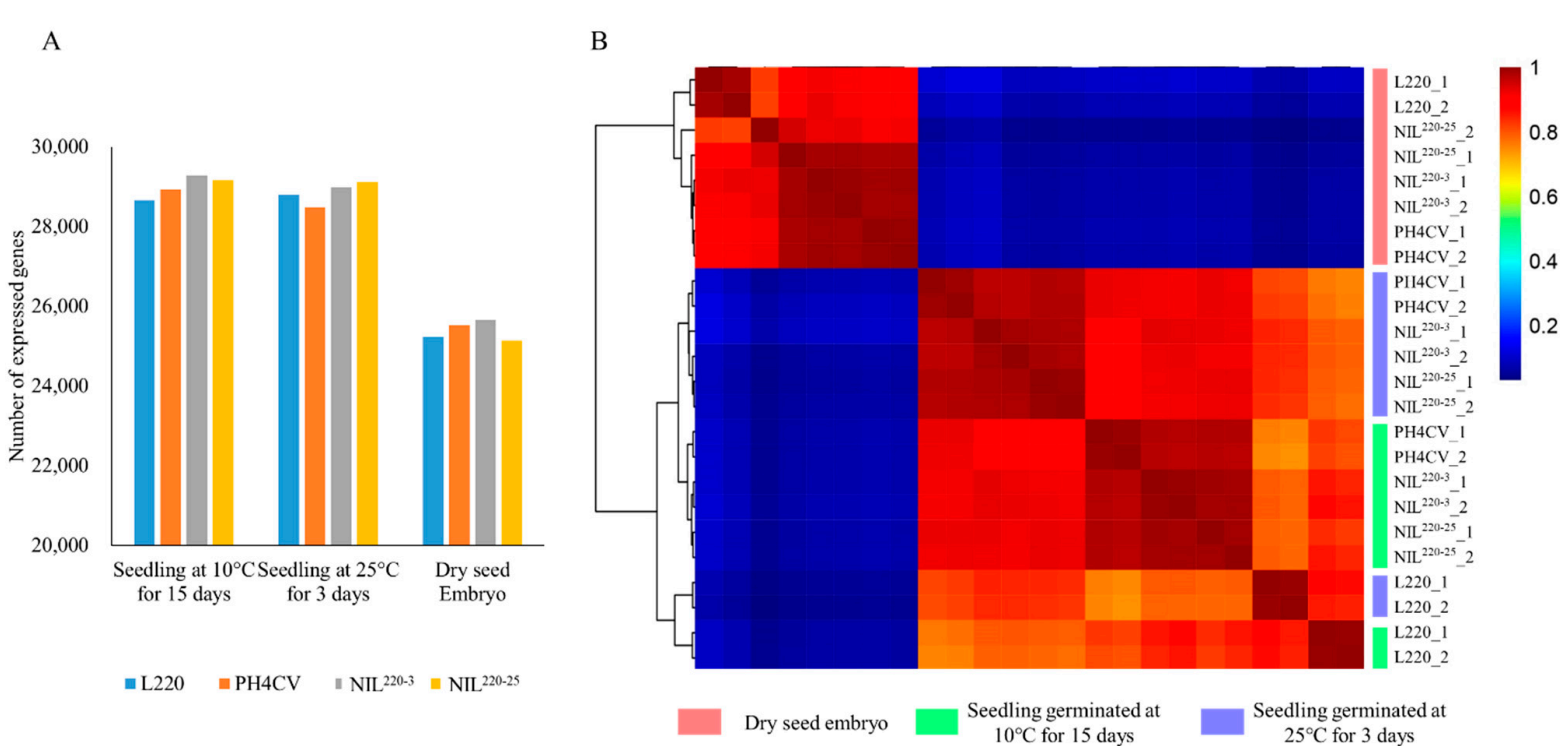
Identification of expressed genes from L220, PH4CV, and their near-isogenic lines NIL^220-3^ and NIL^220-25^. (**A**) The number of expressed genes identified from different genotypes that germinated at 10 °C for 15 days and 25 °C for 3 days, and ungerminated dry embryo. Data were the average of two replications. (**B**) Heatmap clustering of the expressed genes. Pink, green, and blue columns presented vertically at the right side of the heatmap represent samples collected from dry embryo, seedlings germinated at 10 °C for 15 days, and seedlings germinated at 25 °C for 3 days, respectively. Red and blue represent high and low abundance, respectively, according to the normalized FPKM. The numbers 1 and 2 after the genotype represent replicates 1 and 2, respectively.

A total of 24 RNA samples were prepared from CK-0, and CK-3 and Cold-15 of L220, PH4CV, NIL^220-3^, and NIL^220-25^, with two biological replicates. Transcriptome sequencing revealed a total of 563,881,632 clean reads, with reads for each sample ranging from 14,774,469 to 28,918,479 (Table 1). After aligning reads to the maize reference genome (Zm-B73-REFERENCE-GRAMENE-4.0), 79.07–90.82% (mean 88.06%) reads were mapped, with 65.48–81.03% (mean 77.03%) uniquely mapped. When a gene with FPKM (Fragments Per Kilobase of exon model per Million mapped fragments) ≥ 1 was considered as an expressed gene, the number of expressed genes in CK-3 (mean 28,847; range 28,202–29,183) and Cold-15 (mean 29,009; range 28,553–29,349) was similar, which was about 14% more than that in CK-0 (mean 25,387; range 24,732–25,860) (Figure 2A).

Hierarchical cluster analysis was performed to compare gene expression patterns among different samples (Figure 2B), which showed that the two biological replicates clustered closely together, suggesting a good quality transcriptome procedure. Samples from CK-0 were separated from the seedling groups that germinated under either normal (CK-3) or cold (Cold-15) conditions, indicating a large difference in gene expression patterns between dry embryo and germinated seedlings. Within the seedling groups, gene expression patterns of the two NILs showed a closer relationship to the recurrent parent PH4CV than to the donor parent L220, suggesting that the global gene expressions in NILs were more similar to those in PH4CV than in L220, even the LTGA performance in NILs was closer to those in L220 than in PH4CV.

### 2.3. Identification of L220 Specifically Expressed DEGs for LTGA

**Figure 3:**
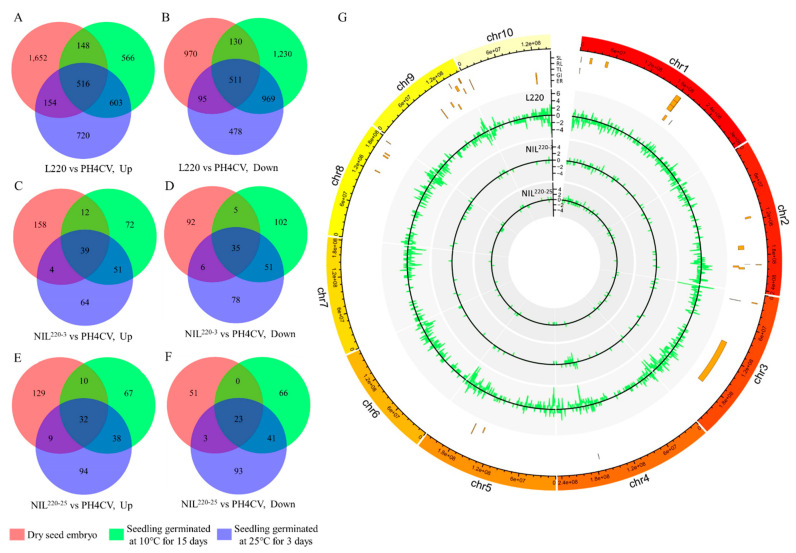
Differentially expressed genes (DEGs) retrieved from L220, NIL^220-3^, and NIL^220-25^ as compared to PH4CV at different germination conditions. (**A**–**F**) Venn diagram illustrating the up- and downregulated DEGs in L220 (**A**,**B**), NIL^220-3^ (**C**,**D**), and NIL^220-25^ (**E**,**F**) as compared to PH4CV at dry seed embryo stage (red cycle), and seedlings germinated at 25 °C for 3 days (blue cycle) and at 10 °C for 15 days (green cycle). (**G**) Distribution of tolerant line (L220, NIL^220-3^, and NIL^220-25^) specifically expressed LTGA genes on the maize genome. Circles from outer to inner showed reference genome (B73, AGPv4), QTLs identified for cold germination in a previous study [31], tolerant line (L220, NIL^220-3^, and NIL^220-25^) specifically expressed gene number in the 1 Mb bin region. Positive or negative numbers indicated up- or downregulated genes.

Compared with the sensitive line PH4CV, different expression genes (DEGs) were screened from tolerant lines L220, NIL^220-3^, and NIL^220-25^ by DEseq2, with the criteria of fold change ≥2 and FDR ≤ 0.001 (Supplementary Table S1). Then, DEGs extracted from Cold-15, but not from CK-0 or CK-3 were defined as LTGA genes. Finally, we revealed 1786 (566 up- and 1230 downregulated), 174 (72 up- and 102 downregulated), and 133 (67 up- and 66 downregulated) LTGA genes from L220, NIL^220-3^, and NIL^220-25^, respectively (Figure 3A–F). DEGs from L220 were distributed across all chromosomes, while most DEGs from the two NILs were located on chromosome 1 (Figure 3G).

**Figure 4:**
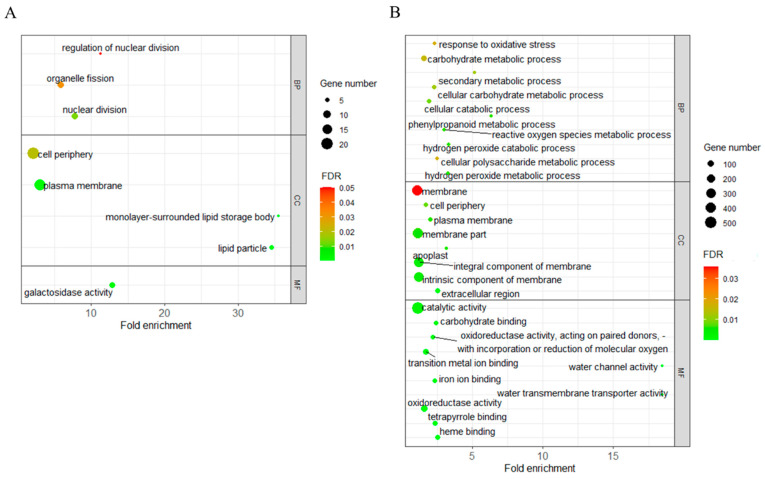
GO enrichment analysis of L220-specifically expressed LTGA genes (DEGs) with up- (**A**) and downregulated (**B**) expression. The size of the dot indicates the number of DEGs involved in the GO category. The color scale indicates the significance level (FDR). The rich factor is the ratio between the number of DEGs and all genes enriched in the category.

GO was a widely used functional classification method for detecting the functional pathway of target traits. Using the AgriGO-v2 software [36], 422 (74.6%) out of the 566 upregulated L220 specifically expressed LGTA genes were assigned into eight GO categories (Figure 4A). Three biological process (BP) related categories (nuclear division, organelle fission, and regulation of nuclear division) were involved in cell division function (Supplementary Table S2). Four categories belonged to the cellular component (CC) aspect, each with two relating to the function of the plasma membrane (plasma membrane and cell periphery) and lipid body (lipid particle and monolayer-surrounded lipid storage body).

GO analyses for the 1230 downregulated L220 specifically expressed LTGA genes revealed 62 GO categories, with 22, 8, and 32 belonging to BP, CC, and MF aspects, respectively (Figure 4B). The top three significant BP categories were associated with reactive oxygen species metabolism. From the eight enriched CC categories, six connected with the plasma membrane (Supplementary Tables S3 and S4), which overlapped with the GO results from the upregulated L220 specifically expressed LTGA genes.

### 2.4. Identification of DEGs Related to qp1ER1-1 Mediated Seed LTGA

**Figure 5:**
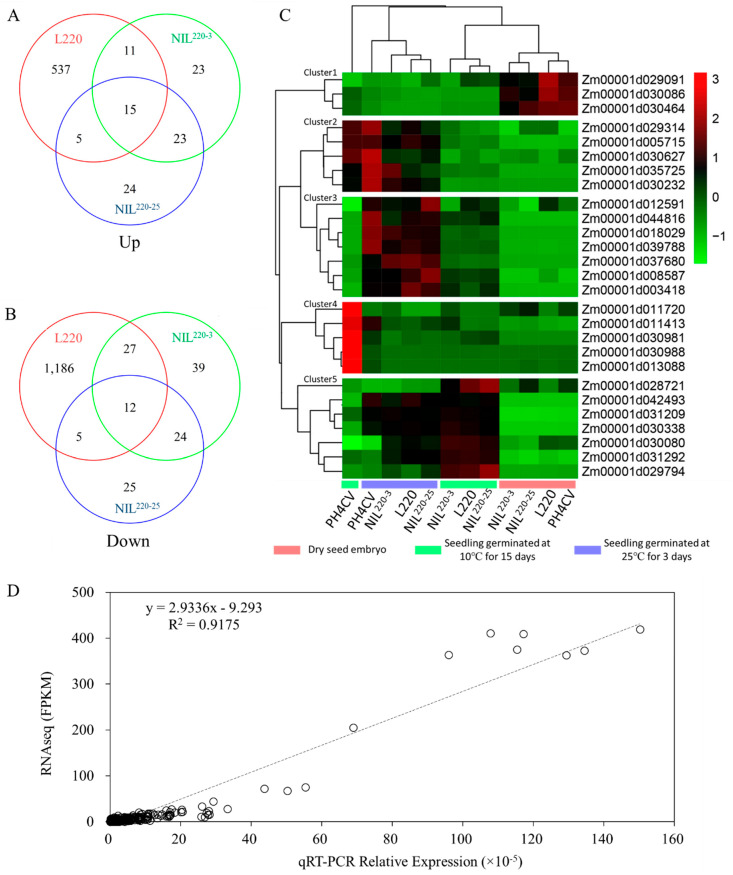
Common DEGs for tolerant line specially expressed LTGA genes. (**A**,**B**) Tolerant line (L220, NIL^220-3^, and NIL^220-25^) specially expressed LTGA genes were identified from Figure 4, which were used for Venn diagram analysis to identify 15 commonly up- (**A**) and 12 downregulated (**B**) genes among the three tolerant genotypes. (**C**) Heat map for the 27 common DEGs. (**D**) qRT-PCR validation of the 27 common DEGs at low and normal germination conditions.

Of the 1796, 174, and 133 genotype specifically expressed LTGA genes identified from L220, NIL^220-3^, and NIL^220-25^, respectively, 27 (15 up- and 12 downregulated) were common LTGA genes that were identified from all three genotypes (Figure 5A,B and Table 2). These genes were defined as *qp1ER1-1*-mediated LTGA genes, because all three genotypes contained *qp1ER1-1* in PH4CV background. Based on gene expression patterns, these genes were clustered into five groups/clusters. Cluster 1 contained 3three genes showing higher expressions in CK-0 than in both CK-3 and Cold-15, indicating a germination suppressive group. Clusters 3 and 5 each had seven genes that showed higher expression in CK-3 than in CK-0, indicating germination inducible groups. Genes in these two groups could increase their expressions in Cold-15 in three tolerant lines but failed to increase in sensitive line PH4CV. Clusters 2 and 4 each contained five genes that showed similar expression levels between Cold-15 and CK-0 in the tolerant lines, but in PH4CV they exclusively increased expression in Cold-15. These results indicated that the increased expression of Cluster 3 and 5 genes and the repressed expression of Cluster 2 and 4 genes might contribute to the increased LTGA in the three tolerant lines.

It is worth noting that Cluster 3 contained three casparian strip membrane genes (CASPs, *Zm00001d018029*, *Zm00001d044816*, and *Zm00001d003418*), which showed upregulated expression in L220 and NILs by comparing with PH4CV. These results suggested important roles of quick membrane repair and cell cycle, contributing to the higher LTGA of L220 and NILs (Table 2).

Integrating the results of transcriptome analysis and QTL mapping was an effective method to predict candidate genes [37]. Our previous work identified 19 QTLs associated with LTGA from the population crossed by L220 and PH4CV [31]. Of the 1786 L220 specifically expressed LTGA genes, 97 (39 up- and 58 downregulated) were located in 13 out of the 19 QTL confidential intervals, with 6 located in the *qp1ER1-1* interval (Figure 3G). Of the 174 NIL^220-3^ and 227 NIL^220-25^ specifically expressed LTGA genes, 11 (6.3%) and 15 (6.6%) were located in QTL confident intervals, with 2 and 3 in the *qp1ER1-1* interval, respectively (Figure 3G). Furthermore, it was interesting to find that three common tolerant line specifically expressed genes were located in QTL regions, with two (*Zm00001d031209* and *Zm00001d031292*) in the *qp1ER1-1* region (Figure 3G).

To verify the reliability of gene expression characterized by transcriptome sequencing, expression of 25 out of the 27 common LGTA genes were qualified by qRT-PCR. All genes showed significantly different expression levels between PH4CV and either L220 or NILs, which is similar to those observed from the RNA-Seq results (Figure 5D).

## 3. Discussion

Dynamic changes in transcript levels occur in maize seedlings at low temperatures, which contributes to plant adaption to environmental changes. In our previous work, we identified a major QTL, *qp1ER1-1*, for the maize LTGA trait [31]. Here, we developed two NILs, NIL^220-3^ and NIL^220-25^, by introgressing the *qp1ER1-1* trait-contributing allele from the high LTGA line L220 into the low line PH4CV. Compared with the recurrent parent PH4CV, both NILs showed significantly increased seedling root and shoot length at Cold-25, while they stayed at the same levels at Cold-15 and CK-3. As mRNA level changes often ahead of phenotype changes in response to environment perturbation [38], we sampled germinated seedlings for RNA sequencing at Cold-15 and CK-3, where all lines showed similar seedling performance, indicating that they stayed at similar developmental stages. This sample harvesting was appropriate for identifying genes in response to low temperature rather than to development difference.

### 3.1. Cell Cycle Participates in Regulating Maize LTGA

**Figure 6:**
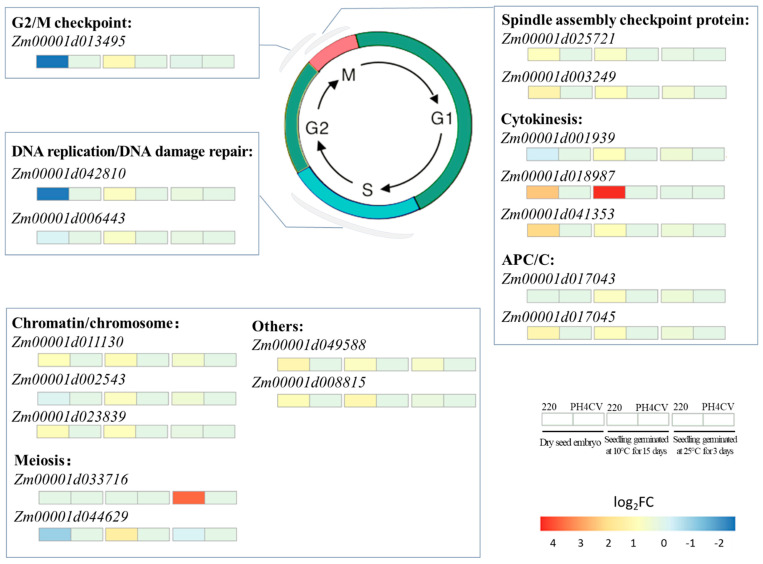
Cell-division-related L220 specifically expressed LTGA DEGs. Red and blue rectangles below the gene name represent up- and downregulated expression in L220 compared with PH4CV.

The method of eukaryotic cell division is mitosis, which would be arrested at G1/S phase by low temperature [39]. Additionally, the expression of genes related to cell cycle control, cell division, and chromosome partitioning changed in response to low temperature, which probably increased plant tolerance to low temperature stress [40]. In this study, 17 cell-division-related genes exhibited higher expression in L220 than in PH4CV under the low temperature germination condition (Figure 6 and Supplementary Table S2). Of them, four genes were involved in cell cycle regulation. *Zm00001d013495* encoded a CDT2 protein, whose homolog in HeLa cells functioned in the early G2/M checkpoint [39,41]. *Zm00001d025721* and *Zm00001d003249* were spindle assembly checkpoint components, homologous to *Mitotic Arrest Deficient 1* (*MAD1*) and *MAD2*, respectively, and played a vital role in preventing anaphase onset before correct attachment of all kinetochores to microtubules and generation of tension [42]. *Zm00001d042810* encoded cell division control protein that was crucial for endoreduplication [43].

As part of the cell division process, cytokinesis was naturally influenced by low temperature [44], and the members of the cytoskeleton participated in plant tolerance to low temperature stress [45]. It suggested that *OsRAN2* might be involved in tubulin transporting and organization of spindles, whose overexpression could improve rice cold tolerance [9,10]. In this work, 3 of the 17 L220 specifically expressed cell division related genes were connected with cytoskeleton, with 2 (*Zm00001d001939* and *Zm00001d018987*) belonging to homolog of tubulin [46,47], and 1 (*Zm00001d041353*) encoding kinesin-like protein KIN-12F that mediated actin-based chloroplast movement in *Arabidopsis* [48]. Together, this work and previous studies suggested that cell-division-related genes were associated with LTGA in maize and indicated that enhancing cell division might be an efficient way to improve plant LTGA.

### 3.2. Plasma Membrane Proteins Participate in Regulating Maize LTGA

At low temperature, the transmembrane transport facilitated the retention of cell osmotic pressure and basic metabolism, which helped cell to acclimate to cold stress [49,50,51]. In the present study, we revealed that DEGs upregulated in both L220 and NILs at low temperature were invariably enriched in plasma-membrane-related GO categories and identified 23 plasma-membrane-related genes (Figure 4, Supplementary Tables S5 and S6). Of them, 14 encoded membrane proteins, with 4 belonging to sugar export transporters (SWEET) that played roles in sugar redistribution. As expression was induced by low temperature, *SWEET* had the potential capacity for low temperature tolerance in plants [52], and their mutation or overexpression would change the sugar concentration and the ability to adapt to low temperature in *Arabidopsis* [52,53,54]. Moreover, overexpression of *AtSWEET16* not only facilitated cold adaption but also promoted seed germination [55]. In addition to *SWEET*, three genes related to inorganic ion transport (Sodium/hydrogen exchanger 4, magnesium transporter NIPA4, H(+)-ATPase11 and sulfate transporter1;3) were also induced by low temperature (Supplementary Table S2). These genes could provide enough nutrients for cell division and seedling growth, which benefited regulation of cytosolic concentration during seed germination. Thus, promoting the transmembrane transport process would be beneficial for cold acclimation in plants.

We were surprised to find four membrane-protein-related genes encoding CASP proteins, whose transcript levels were specifically upregulated in L220 at low temperature. Low-temperature-induced expression of CASP genes was also found in *Arabidopsis* [56]. In addition, knock-out of *AtCASPL4C1* elevated tolerance to cold stress, and overexpressing *CICASPL* resulted in increased sensitivity to cold stress in *Arabidopsis* [56]. In maize, *ZmCASP2a1* served as a candidate gene for a QTL of seed LTGA [57]. All these results suggested an important role of *CASP/CASPL* genes in plant adaptation to low temperatures.

In plants, CASP could mark a special domain on the membrane where an area of Casparian strips was predicted by recruiting the lignin polymerization machinery [58]. Since Casparian strips could establish a barrier between the symplast and apoplast, it was speculated that CASPs were associated with nutrient transporting indirectly, but further studies were required to uncover the detailed function of CASP in seed LTGA.

### 3.3. Transcriptome Analysis of NILs for Predicting Genes Related to qp1ER1-1

Two NILs were used to narrow down the number of DEGs and predict candidate causal genes for *qp1ER1-1*. Compared to the recurrent parent PH4CV, the ER and seedling TL, RL, and SL were significantly improved in NIL_220-3_ and NIL_220-25_ that carried the tolerant allele of *qp1ER1-1* in PH4CV genetic background (Figure 1). A total of 27 genes (15 upregulated and 12 downregulated) were found commonly expressed in NILs and L220 at low temperature (Figure 5). Two commonly upregulated genes (*Zm00001d031209* and *Zm00001d031292*) were in the confidence interval of *qp1ER1-1* (Supplementary Table S1), which could be assumed as the causal genes underlying *qp1ER1-1*. *Zm00001d031209* encoded DIMBOA UDP-glucosyltransferase BX9 (benzoxazinone synthesis 9) and involved in benzoxazinoid synthesis [59], whose enzyme activity was upregulated in maize leaf under drought conditions [60]. *Zm00001d031292* encoded a harpin-induced protein, belonging to the late embryogenesis abundant (LEA) hydroxyproline-rich glycoprotein family protein. Previous work showed that expression of its homolog in *Chorispora bungeana* seedlings was induced by chilling treatment [61]. Additionally, overexpressing *MeDREB1D(R-2)* and *MeDREB1D(Y-3)* in *Arabidopsis* resulted in stronger tolerance to cold stress by accompanying increased expression of two *Zm00001d031292* homologs [62]. Taken together, these works suggested that *Zm00001d031209* and *Zm00001d031292* had the possibility to associate with *qp1ER1-1* improved seed LTGA in maize.

## 4. Materials and Methods

### 4.1. Plant Materials

Maize inbred lines L220 and PH4CV were collected as high and low seed LTGA, respectively, which have been described in our previous study [31]. Two near isogenic lines (NIL^220-3^ and NIL^220-25^) were screened from more than 200 BC_4_F_4_ individuals that were obtained by backcrossing an inbred line L220 to PH4CV. The molecular markers umc1754 (F: ATAGGGATCGACCCGTTCGT, R: AATATCTCCGATCCACCAACAAAA) and indel8 (F: GTGTTAAGACCCACTGCGTC, R: GCACGGCATCCCATGTAATT) were used for the marker-assisted backcrossing procedure. A 6k SNP chip (Illumina Inc., San Diego, CA, USA) was adopted to characterize the genetic background of NIL^220-3^ and NIL^220-25^, according the procedure published in our previous work [31]

### 4.2. Emergence Rate and Seedling Morphological Traits’ Evaluation at Low Temperature

Seed harvested from L220, PH4CV, and NILs were used for LTGA evaluation according to the methods described by Li et al. (2018) and Zhang et al. (2020) [31,35]. For emergence rate testing, 30 seeds were surface sterilized for 5 min in 0.1% sodium hypochlorite and rinsed three times with distilled water, then sown in sterile sand with moisture content of 16% in a plastic box, which incubated in a dark chamber at 10 °C ER was defined as shoots breaking through the sand and counted from 17 to 25 days after sowing (DAS) with a 2-day interval. For seedling morphological trait evaluation, 10 sterilized seeds were sown in fully moist germination paper (Anchor Ltd., St. Paul, MN, USA), then the paper was rolled vertically in a sealed plastic bag, and the paper rolls were cultured in a 10 °C chamber. Seedling TL, RL, and SL were measured with a ruler at 15 and 25 DAS, and average of the 10 seedlings served as the trait value for each line. Seedling performance at normal temperature was measured at 3 DAS after culture at 25 °C. All lines were tested with three replicates.

### 4.3. Sample Preparation, Transcriptome Sequencing, and Data Analysis

Seedlings germinated at Cold-15 and CK-3 in paper rolls were harvested and used for transcriptome analysis with two replicates. Embryos of dry seeds were used as an additional control. Ten seedlings or embryos in each replication were pooled and ground in liquid nitrogen for total RNA extraction using RNAprep pure Plant Kit (Tiangen Biotech, Beijing, China). A total of 1.5 μg cleaned RNA per sample was used as input material for sequencing library construction. Sequencing libraries were generated using NEBNext UltraTM RNA Library Prep Kit for Illumina (New England Biolabs, Ipswich, MA, USA) following the manufacturer’s recommendations. The libraries were then sequenced on an Illumina platform and 150 bp paired-end reads were generated.

The raw reads were filtered and trimmed to remove adaptors and low quality reads via FastQC and Trimmomatic, respectively, with parameters: LEADING:3, TRAILING:3, SLIDINGWINDOW:4:15; MINLEN:36; MAXINFO:40:0.9; and HEADCROP:7 [63]. Subsequently, clean reads were aligned to the B73 reference genome (Zm-B73-REFERENCE-GRAMENE-4.0) by hisat2 [64,65]. A read counts matrix was generated using Subread/featureConuts with the default parameters [66]. FPKM was used for indexing gene expression abundance, which was further used for plotting heatmap figures using the pheatmap R package (v1.0.12). DESeq2 (R package) was adapted to quantify gene expression and identify DEGs with threshold of adjust *p* < 0.001 and |log2-FoldChange| > 1, and the adjust *p* values were calculated using the Benjamini and Hochberge method. Venn diagrams were drawn by VennDiagram.

Gene Ontology (GO) enrichment analyses were performed by web-based software AgriGO-v2 at http://systemsbiology.cau.edu.cn/agriGOv2/ (accessed on 23 February 2019) [34]. Significant GO categories were identified using a cutoff FDR < 0.05, which were divided into biological processes, molecular function, and subcellular locations. The annotation of each gene was obtained from the National Center for Biotechnology Information (NBCI, https://www.ncbi.nlm.nih.gov/gene/ (accessed on 9 March 2019)), which was verified at maizeGDB (www.maizegdb.org (accessed on 12 March 2022)) (Supplementary Table S7).

### 4.4. qRT-PCR Validation of RNAseq Data

To validate the gene expression level from RNA sequencing, 27 commonly expressed genes among L220 and NILs at low temperature were subjected to quantitative real-time PCR analysis using QuantStudio 6 (Thermo Fisher, Waltham, MA, USA). The maize *ZmGAPDH* served as an endogenous control. cDNA was synthesized from the RNA prepared for RNA sequencing. Primers used for qRT-PCR are listed in Supplementary Table S8. Three biological replicates were conducted and each biological replicate was technically repeated three times. The 2^−ΔCT^ method was used to calculate gene transcript relative abundance [67].

## 5. Conclusions

By comparing the transcriptomes of two maize inbred lines (L220 and PH4CV), the cell division process and plasma membrane proteins were found to closely relate to maize seed LTGA. We speculated that high expression of membrane protein related genes could help to provide enough nutrients for cell division and seedling growth, which subsequently improve seed germination under cold condition. By incorporating two NILs into the analysis, a total of 27 genes were identified as tolerant line (L220 and two NILs) specifically expressed LTGA genes. Two (*Zm00001d031209* and *Zm00001d031292*) of the upregulated genes were located in the confidence interval of *qp1ER1-1*. Collectively, even RNAseq itself had potential limitation for causal gene identification; these findings could guide the isolation of the putative candidate gene underlying *qp1ER1-1* and provide insight into the mechanism of maize seed LTGA.

## Figures and Tables

**Table 1:** Summary of transcriptome sequencing.

Lines	Rep	Total Reads	Rate of Total Mapped Reads (%)	Rate of Uniquely Mapped Reads (%)	Num. of Expressed Genes	Rate of Expressed Genes (%)
Seedlings germinated at 10 °C for 15 days						
L220	1	24,027,959	85.81	73.47	28,758	73.14
	2	21,709,642	85.81	73.51	28,553	72.62
PH4CV	1	27,815,147	86.04	74.13	28,852	73.38
	2	23,897,333	85.77	73.83	29,023	73.81
NIL^220-3^	1	25,654,608	85.36	73.38	29,349	74.64
	2	25,184,639	79.07	65.48	29,211	74.29
NIL^220-25^	1	22,445,754	85.60	73.97	29,025	73.82
	2	22,072,033	84.45	72.31	29,298	74.51
Seedlings germinated at 25 °C for 3 days						
L220	1	21,186,558	89.75	79.59	28,827	73.31
	2	27,799,551	89.58	78.79	28,756	73.13
PH4CV	1	25,034,433	89.45	78.60	28,202	71.72
	2	22,058,337	88.82	77.78	28,787	73.21
NIL^220-3^	1	23,740,395	90.31	80.27	28,888	73.47
	2	23,529,687	89.12	78.53	29,072	73.94
NIL^220-25^	1	17,003,680	89.76	79.70	29,062	73.91
	2	18,777,146	89.44	79.26	29,183	74.22
Dry seed embryo						
L220	1	22,004,941	90.05	80.04	25,352	64.48
	2	23,104,023	88.92	78.28	25,110	63.86
PH4CV	1	14,774,469	89.99	79.85	25,568	65.03
	2	23,542,315	89.46	78.43	25,486	64.82
NIL^220-3^	1	25,681,524	89.54	78.57	25,860	65.77
	2	28,918,479	89.73	78.99	25,447	64.72
NIL^220-25^	1	25,480,625	90.76	80.96	25,538	64.95
	2	28,438,354	90.82	81.03	24,732	62.90

**Table 2:** Common DEGs for tolerant line (L220, NIL3, and NIL25) specially expressed cold germination genes.

Gene	Position	Description	Cluster	Up/Down
Zm00001d012591	Chr8:176955466-176958213	protein disulfide isomerase	Cluster 3	Up
Zm00001d029091	Chr1:57485976-57493258	sucrose synthase 2	Cluster 1	Up
Zm00001d031209	Chr1:182293107-182294953	bx9-benzoxazinone synthesis9	Cluster 5	Up
Zm00001d031292	Chr1:185477807-185478616	harpin-induced protein	Cluster 5	Up
Zm00001d042493	Chr3:169439385-169441492	extensin	Cluster 5	Up
Zm00001d037680	Chr6:133844417-133848578	Vegetative storage protein 2	Cluster 3	Up
Zm00001d018029	Chr5:212390087-212391281	casparian strip membrane protein 3	Cluster 3	Up
Zm00001d008587	Chr8:13681436-13683379	Class I glutamine amidotransferase-like superfamily protein	Cluster 3	Up
Zm00001d030338	Chr1:123276181-123277143	expressed protein	Cluster 5	Up
Zm00001d030080	Chr1:104119711-104120879	NADH dehydrogenase ubiquinone 1 beta subcomplex subunit 10-A	Cluster 5	Up
Zm00001d029794	Chr1:87314979-87316146	Basic endochitinase B	Cluster 5	Up
Zm00001d044816	Chr9:3595060-3596502	casparian strip membrane protein 1	Cluster 3	Up
Zm00001d028721	Chr1:44340623-44341737	lbd4-LBD-transcription factor 4	Cluster 5	Up
Zm00001d039788	Chr3:14747663-14748751	dirigent-like protein	Cluster 3	Up
Zm00001d003418	Chr2:43780271-43781507	casparian strip membrane protein 4	Cluster 3	Up
Zm00001d011720	Chr8:159835548-159840848	violaxanthin de-epoxidase3	Cluster 4	Up
Zm00001d029314	Chr1:65618909-65621774	cationic amino acid transporter	Cluster 2	Down
Zm00001d011413	Chr8:149844919-149847262	wrky28-WRKY-transcription factor 28	Cluster 4	Down
Zm00001d005715	Chr2:184452989-184461356	vacuolar iron transporter 1.2	Cluster 2	Down
Zm00001d035725	Chr6:43572975-43574070	late embryogenesis abundant protein-related/LEA protein-related	Cluster 2	Down
Zm00001d030086	Chr1:104416569-104417982	Tetratricopeptide repeat (TPR)-like superfamily protein	Cluster 1	Down
Zm00001d030988	Chr1:172180389-172181046	expressed protein	Cluster 4	Down
Zm00001d013088	Chr5:4532993-4533650	Cytochrome P450	Cluster 4	Down
Zm00001d030627	Chr1:149688164-149696743	prefoldin subunit 3	Cluster 2	Down
Zm00001d030981	Chr1:171564539-171568092	probable LRR receptor-like serine/threonine-protein kinase	Cluster 4	Down
Zm00001d030464	Chr1:134521008-134522680	carbohydrate transporter/sugar porter	Cluster 1	Down
Zm00001d030232	Chr1:114928784-114930818	bhlh111–bHLH-transcription factor 111	Cluster 2	Down

## Data Availability

The data that support the findings of this study are available at NCBI under SRA accession number PRJNA759333.

## Supplementary Materials

The following supporting information can be downloaded at: https://www.mdpi.com/article/10.3390/plants11070887/s1, Table S1. DEGs detected in this study; Table S2. L220 specifically expressed LTGA DEGs relating to cell cycle, lipid and plasma membrane functions; Table S3. Plasma membrane associated GO categories enriched from the L220 specifically down-regulated LTGA DEGs; Table S4. Plasma membrane associated L220 specifically down-regulated LTGA DEGs; Table S5. Significant GO categories for NIL^220-3^ specifically expressed DEGs; Table S6. Significant GO categories for NIL^220-25^ specifically expressed DEGs; Table S7. The annotation of the 27 common tolerant specific DEGs in MaizeGDB database; Table S8. Primers used in this work.
